# A ghost imaging framework based on laser mode speckle pattern for underwater environments

**DOI:** 10.1038/s44172-024-00200-9

**Published:** 2024-03-21

**Authors:** Mo-Chou Yang, Peng Wang, Yi Wu, Guo-Ying Feng

**Affiliations:** Institute of Laser & Micro/Nano Engineering, College of Electronics and Information Engineering, Sichuan University, Chengdu, Sichuan 610065 China

**Keywords:** Imaging and sensing, Information technology

## Abstract

Due to the complex physical processes found in underwater environments, such as absorption, scattering, and noise, it is challenging to obtain high-quality images using conventional camera-based imaging techniques. Ghost imaging possesses strong anti-interference capabilities and can effectively obtain images in underwater environments. Here, we propose a ghost imaging framework based on a physical model of M^2^-ordered laser mode patterns and apply it to Ghost Imaging. The simulation results show that the Laser Mode Speckle Ghost Imaging can reconstruct the overall trapped contour even at a low sampling rate, specifically below 0.64%. A high-quality image with a Peak Signal-to-Noise Ratio of 19 dB can be achieved using the Laser Mode Speckle Ghost Imaging when the sampling rate is 5%. Even with a relative random noise of 1.0%–5.0%, the imaging quality of Laser Mode Speckle Ghost Imaging is superior to that of Random speckle pattern Ghost Imaging, Walsh speckle pattern Ghost Imaging, and Haar speckle pattern Ghost Imaging when the sampling rate consistent. Our experimental results in a turbid water environment confirm the conclusions drawn from the simulation results. The proposed Laser Mode Speckle Ghost Imaging can be used as an imaging solution in challenging liquid environments, such as turbid liquids, inclement weather, and biological tissue fluids.

## Abstract (web summary)

Mochou Yang and colleagues describe a a ghost imaging framework based on laser mode speckle pattern which improves imaging quality at low sampling rate. The feasibility of this method is demonstrated in a turbid water environment.

## Introduction

**Fig. 1:**
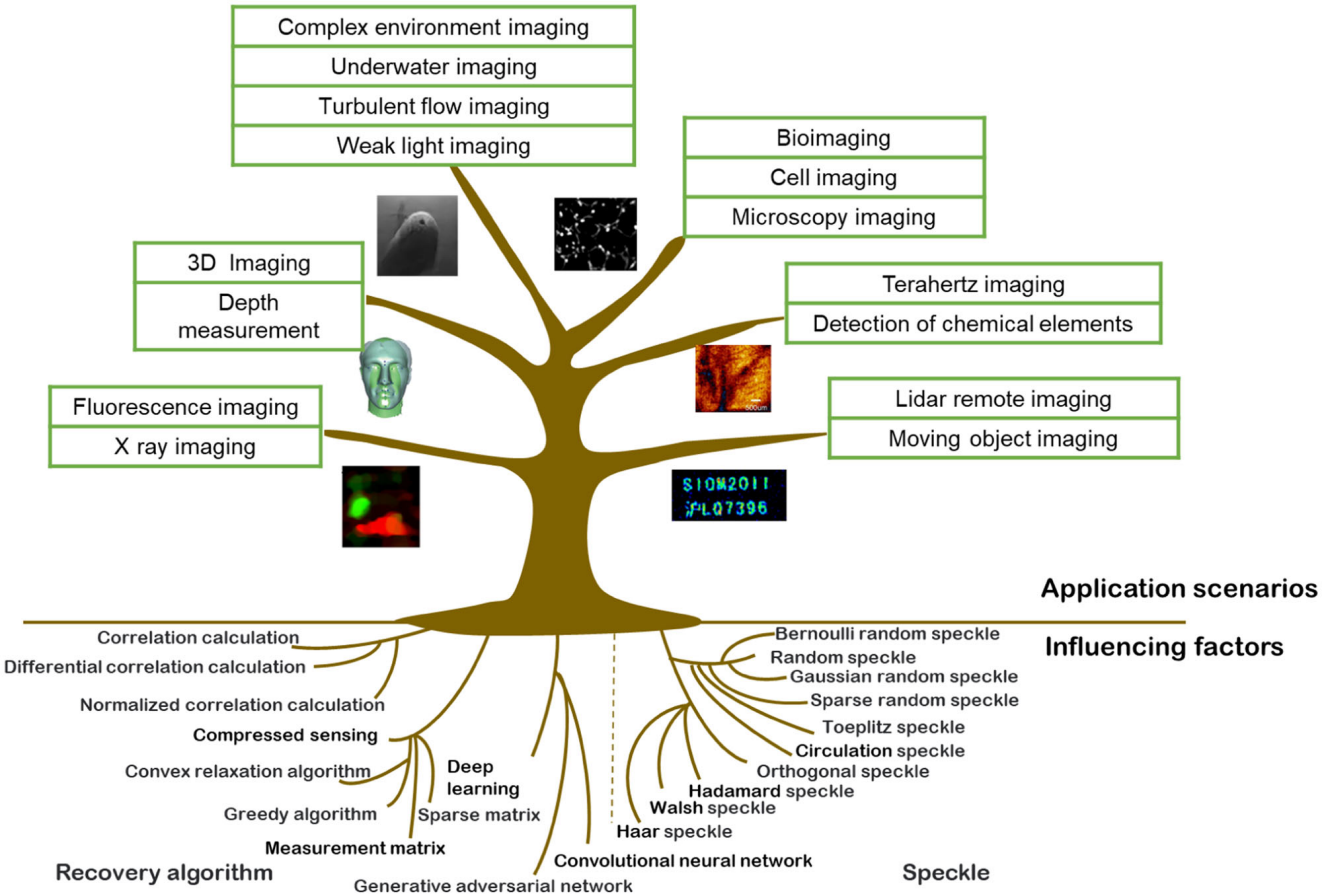
Schematic diagram of influencing factors and application scenarios of ghost imaging (GI). The influencing factors mainly include the speckle patterns and recovery algorithm, and the application scenarios mainly include 3D imaging^5^, fluorescence imaging^1^, lidar^29^, terahertz imaging^3^, bioimaging^30^, and underwater imaging^10^.

When performing target detection in underwater environments, the complexity of the underwater environment, including physical factors such as absorption, scattering, and noise, as well as the low sensitivity of the detector, results in low imaging quality when using conventional camera-based imaging techniques. As a result, there are greater difficulties in discovering and recognizing objects in these environments. Ghost imaging (GI) technology is a modern method that can produce high-quality images in complex environments, and it relies on the illumination speckle pattern for its realization. In GI, objects are illuminated with speckle patterns, which are subsequently collected by a bucket detector without spatial resolution. These patterns are used to form a reconstructed image through correlation calculation. Owing to its wide range of light sources and strong anti-interference ability, GI has attracted significant attention in several related fields such as X-ray imaging^1,2^, terahertz imaging^3^, acoustic imaging^4^, three-dimensional imaging^5,6^, fluorescence imaging^1^, optical encryption^7,8^, and underwater imaging^9–15^. The application scenarios and influencing factors of GI are depicted in Fig. 1.

Recently, researchers have discussed the effect of speckle patterns on GI^16,17^. According to mathematical distribution, modulated speckle patterns can be classified into random and orthogonal types. Scholars have utilized various random speckle patterns in GI, including Gaussian random type^18^, Bernoulli random type^19^, pink noise type^20^, and orthogonal speckle patterns such as the Fourier type^21^, Hadamard type^22^, Walsh type^23^, rearranged Hadamard type^24^, Hadamard–Haar type^25^, “average sparsity”^26^, and cosinusoidal-encoding multiplexed type^27,28^. Typically, random speckle patterns should satisfy the Nyquist theorem when performing correlation calculations. In other words, effective imaging can only be achieved when the sampling rate is two. However, orthogonal speckle patterns can achieve perfect imaging when the sampling rate is one. These speckle patterns typically necessitate a higher sampling rate to achieve better imaging quality, which results in a significant time consumption when excluding non-interested targets. The efficiency of detecting interested targets in complex underwater environments still needs improvement.

Here, we propose a ghost imaging framework based on the physical model of *M*^2^-ordered laser mode patterns and adopt the GI method for underwater monitoring complex environments. Laser mode speckle pattern GI (LMS-GI) can quickly capture most of the information from unknown targets at a low sampling rate, filter out irrelevant targets, and then perform imaging on the targets of interest. This method can significantly improve the speed of detecting the targets of interest in complex underwater environments. Furthermore, LMS-GI is highly robust and outperforms conventional GI in real underwater environments. In highly turbid underwater environments, it can achieve low-sampling imaging comparable to that obtained using compressed sensing and deep learning. In the future, LMS-GI could be extensively utilized in remote sensing, bioimaging, and other fields because of its low sampling rate and high imaging quality.

## Results

### Laser modes

**Fig. 2:**
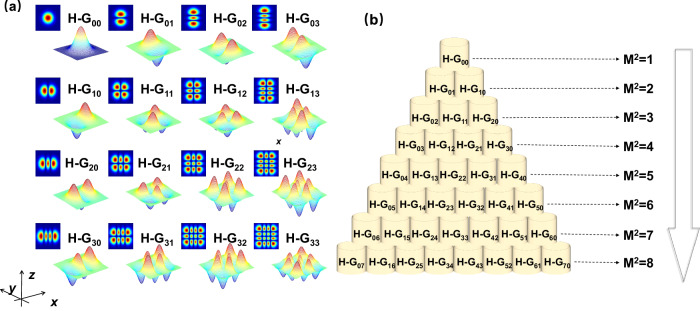
Hermitian-Gaussian (H–G) _00_ ~ H–G_33_ modes and their sorting by *M*_*mn*_^2^ values. **a** The two-dimensional intensity distributions and three-dimensional complex amplitude distributions of H–G_00_ ~ H–G_33_ modes. **b** H–G_*mn*_ laser modes are sorted according to their *M*_*mn*_^2^ values.

In a Cartesian coordinate system $(x,y,z)$, the paraxial Helmholtz equation is:

1 $$\frac{{\partial }^{2}E}{\partial {x}^{2}}+\frac{{\partial }^{2}E}{\partial {y}^{2}}+2{{{{{\rm{i}}}}}}k\frac{\partial E}{\partial z}=0$$

where $E(x,y,z)$ is the slow-varying amplitude of the electric field, ${k}^{2}={k}_{0}^{2}{n}^{2}$, ${k}_{0}=2{{{{{\rm{\pi }}}}}}/\lambda$ is the number of the waves in a vacuum,$\lambda$ is the wavelength, and *n* is the refractive index of the medium. The solutions to the paraxial wave equation in Cartesian coordinates are the Hermitian-Gaussian (${{{{{\rm{H}}}}}}-{{{{{\rm{G}}}}}}$) laser modes with an orthogonal unification completeness, referred to as ${{{{{{\rm{H}}}}}}-{{{{{\rm{G}}}}}}}_{mn}$ mode. For a square spherical mirror symmetrical confocal cavity with a side length of 2*a* and a cavity length of $L$, the mathematical expression of a ${{{{{{\rm{H}}}}}}-{{{{{\rm{G}}}}}}}_{mn}$ laser mode is as below,

2 $${E}_{mn}(x,y)={C}_{mn}{{{{{{\rm{H}}}}}}}_{m}\left(\sqrt{\frac{2{{{{{\rm{\pi }}}}}}}{L\lambda }}x\right){{{{{{\rm{H}}}}}}}_{n}\left(\sqrt{\frac{2{{{{{\rm{\pi }}}}}}}{L\lambda }}y\right){{{{{{\rm{e}}}}}}}^{-\frac{{x}^{2}+{y}^{2}}{L\lambda /{{{{{\rm{\pi }}}}}}}}$$

where *m* and *n* are indices of laser modes. *m* + 1 represents the number of nodes in the *x* direction, and *n* + 1 represents the number of nodes in *y* direction. *C*_*mn*_ is the normalized constant of the H–G_*mn*_ mode. H_*m*_ and H_*n*_ are the *m*-th and *n*-th order Hermite polynomials, respectively.

3 $$\begin{array}{c}{{{{{{\rm{H}}}}}}}_{m}(X)=\mathop{\sum }\limits_{k=0}^{[\frac{m}{2}]}\frac{{(-1)}^{k}m!}{k!(m-2k)!}{(2X)}^{m-2k}\\ {{{{{{\rm{H}}}}}}}_{n}(Y)=\mathop{\sum }\limits_{k=0}^{[\frac{n}{2}]}\frac{{(-1)}^{k}n!}{k!(n-2k)!}{(2Y)}^{n-2k}\end{array}$$

where $[\frac{m}{2}]$ is the integer part of $\frac{m}{2}$, $[\frac{n}{2}]$ is the integer part of $\frac{n}{2}$, $X=\frac{\sqrt{c}}{a}x$,$Y=\frac{\sqrt{c}}{a}y$, $c=2{{{{{\rm{\pi }}}}}}N$, and the Fresnel number $N={a}^{2}/(\lambda L)$. The two-dimensional intensity distributions and three-dimensional complex amplitude distributions of H–G_00_ to H–G_33_ laser modes are shown in Fig. 2a.

Siegman introduced the concepts of spatial frequency and intensity moment while establishing a relatively complete theoretical model for the concept of the *M*^2^ factor used to evaluate laser beam quality^29–31^. The *M*^2^ factor has become one of the most widely accepted parameters for evaluating the beam quality of laser beams^32–35^. The waist radius and the far-field divergence half-angle of the fundamental Gaussian H–G_00_ laser mode are set as *w*_0_ and *θ*_0_, and

4 $${w}_{0}{\theta }_{0}=\frac{\lambda }{{{{{{\rm{\pi }}}}}}}$$

The waist radii of the H–G_*mn*_ laser mode in the *x* direction, *y* direction and the *r* radial direction (${r}^{2}={x}^{2}+{y}^{2}$, which represents the square of the distance of a point $(x,y)$ from the propagation axis *z*), are:

5 $$\begin{array}{c}{w}_{0x,mn}^{2}=(2m+1){w}_{{{{{{\rm{o}}}}}}}^{2}\\ {w}_{0y,mn}^{2}=(2n+1){w}_{{{{{{\rm{o}}}}}}}^{2}\end{array}$$

The waist radii of the ${{{{{{\rm{H}}}}}}-{{{{{\rm{G}}}}}}}_{mn}$ laser mode in the *r* radial direction is:

6 $${w}_{0r,mn}^{2}=2(m+n+1){w}_{{{{{{\rm{o}}}}}}}^{2}$$

The far-field diverging half-angles of the H–G_*mn*_ laser mode in the *x* direction, *y* direction, and *r* radial direction are:

7 $${\theta }_{x,mn}=	\,\sqrt{2m+1}{\theta }_{0}\\ {\theta }_{y,mn}=	\,\sqrt{2n+1}{\theta }_{0}\\ {\theta }_{r,mn}=	\,\sqrt{(2m+1)(2n+1)}{\theta }_{0}$$

The *M*^2^ factors of H–G_*mn*_ laser mode in the *x* and *y* directions can be expressed as:

8 $$\begin{array}{c}{M}_{x}^{2}=2m+1\\ {M}_{y}^{2}=2n+1\end{array}$$

The *M*^2^ factors of H–G_*mn*_ laser beam in the *r* radial direction can be expressed as:

9 $${M}_{r}^{2}=m+n+1$$

The H–G_*mn*_ laser modes are sorted according to their beam quality *M*_*mn*_^2^ values from 1 to 8, as shown in Fig. 2b. The larger the value of *M*_*mn*_^2^ factor, the larger the sequential number of H–G_*mn*_ laser modes included. The increase in the sequential number of laser modes is characterized by a ladder shape. The fundamental laser mode H–G_00_ (*m* = *n* = 0) represents a zero spatial frequency signal, while the higher order laser mode H–G_*mn*_ ($m\, \ne \, 0$ or $n\, \ne \, 0$) represents a higher spatial frequency signal.

### Ghost imaging model

**Fig. 3:**
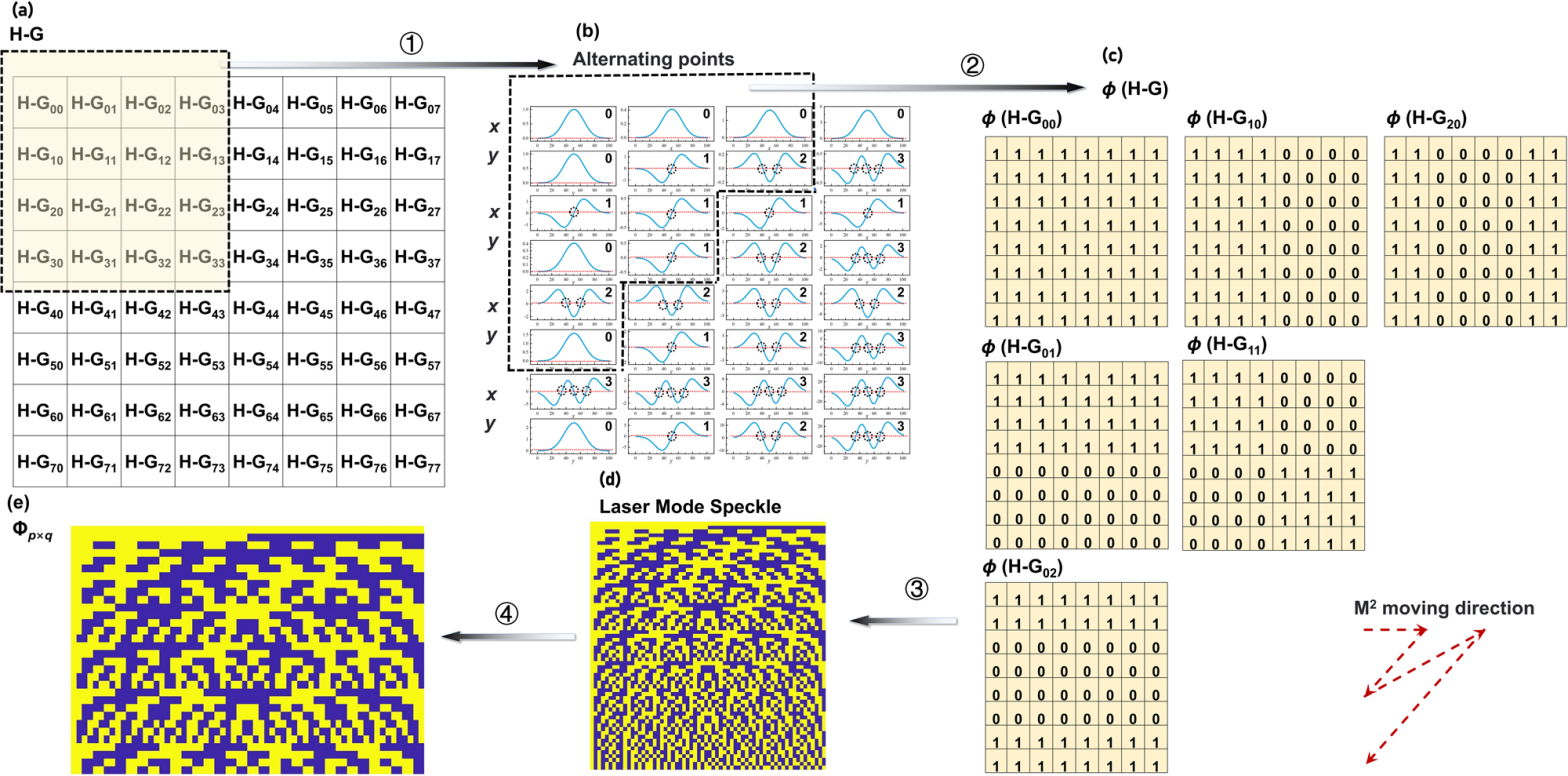
Formation process of laser mode speckle patterns. **a** Structure diagram of Hermitian-Gaussian (H–G)_00_ ~ H–G_77_ mode. **b** Zero change point of amplitude in H–G_00_ ~ H–G_33_ mode. **c** Checkerboard pattern of the two-phase values 0 and 1. for *M*_*mn*_^2^ ≤ 3. **d** Complete laser speckle patterns at an 8×8 resolution. **e** Part of laser mode speckle patterns at an 8 × 8 resolution.

Figure 3 depicts the formation of LMS with a solution of 8 × 8 pixels. Figure 3a shows the matrix distribution of 64 modes (H–G_00_ ~ H–G_77_). 16 modes (H–G_00_ ~ H–G_33_) are selected for depicting numerically simulated amplitude and phase in the *x* and *y* directions, as shown in Fig. 3b. Adjacent nodes have a phase shift of π. Phase patterns from standard H–G beams were simulated, and checkerboard patterns $\varphi ({{{{{{\rm{H}}}}}}-{{{{{\rm{G}}}}}}}_{mn}),m=1,{{{{\mathrm{.}}}}}..,{q}_{1},n=1,{{{{\mathrm{.}}}}}..,{q}_{2}$, two-phase values (0 and 1) based on laser modes were obtained, as shown in Fig. 3c. The speckle patterns of H–G_*mn*_ laser modes were sorted according to their *M*_*mn*_^2^ values, and a complete speckle pattern matrix was obtained, as shown in Fig. 3d. Part of the speckle pattern matrix is obtained after under-sampling, as shown in Fig. 3e. The$\varphi ({{{{{{\rm{H}}}}}}-{{{{{\rm{G}}}}}}}_{mn}),m=1,{{{{\mathrm{.}}}}}..,{q}_{1},n=1,{{{{\mathrm{.}}}}}..,{q}_{2}$ were converted to *q* dimensional data. The corresponding speckle pattern matrix is as follows:

10 $$\varPhi =\left[\begin{array}{c}\varphi ({{{{{{\rm{H}}}}}}-{{{{{\rm{G}}}}}}}_{00})\\ \varphi ({{{{{{\rm{H}}}}}}-{{{{{\rm{G}}}}}}}_{01})\\ \vdots \\ \varphi ({{{{{{\rm{H}}}}}}-{{{{{\rm{G}}}}}}}_{{q}_{1}{q}_{2}})\end{array}\right]$$

### Imaging quality standards

We used Peak Signal-to-Noise Ratio (PSNR) and Structural Similarity (SSIM) to characterize the image quality of the GI image $G(x,y)$ which is relative to the object $T(x,y)$ being measured. The PSNR^36^ is:

11 $$PSNR=10{\log }_{10}\left(\frac{{q}_{1}{q}_{2}\cdot {{{{{{\rm{MAX}}}}}}}^{2}}{\mathop{\sum }\nolimits_{x=1}^{{n}_{1}}\mathop{\sum }\nolimits_{y=1}^{{n}_{2}}{[G(x,y)-T(x,y)]}^{2}}\right)$$

where MAX represents the maximum possible pixel value of the image; in this study, MAX = 255.

The SSIM^37^ is:

12 $$SSIM(G,T) =	 L(G,T)\ast C(G,T)\ast S(G,T)\\ L(G,T) =	\frac{2{u}_{G}{u}_{T}+{C}_{1}}{{{u}_{G}}^{2}+{{u}_{T}}^{2}+{C}_{1}}\\ C(G,T) = 	\frac{2{\sigma }_{G}{\sigma }_{T}+{C}_{2}}{{{\sigma }_{G}}^{2}+{{\sigma }_{T}}^{2}+{C}_{2}}\\ S(G,T) =	 \frac{{\sigma }_{GT}+{C}_{3}}{{\sigma }_{G}{\sigma }_{T}+{C}_{3}}$$

where ${u}_{G}$ and ${u}_{T}$ are the averages of $G(x,y)$ and the object $T(x,y)$, respectively. ${\sigma }_{G}$ and ${\sigma }_{T}$ are the standard deviations of the GI and the object, respectively, and ${\sigma }_{GT}$ is the covariance between the GI and the object. *C*_1_, *C*_2_, and *C*_3_ are constants used to guarantee that the denominator of the formula is not zero. In general, ${C}_{1}={({K}_{1}{L}_{1})}^{2}$,${C}_{2}={({K}_{2}{L}_{1})}^{2}$, ${C}_{3}={C}_{2}/2$, ${K}_{1}=0.01$,${K}_{2}=0.03$, and$\,{L}_{1}=255$.

### Experiment setup

**Fig. 4:**
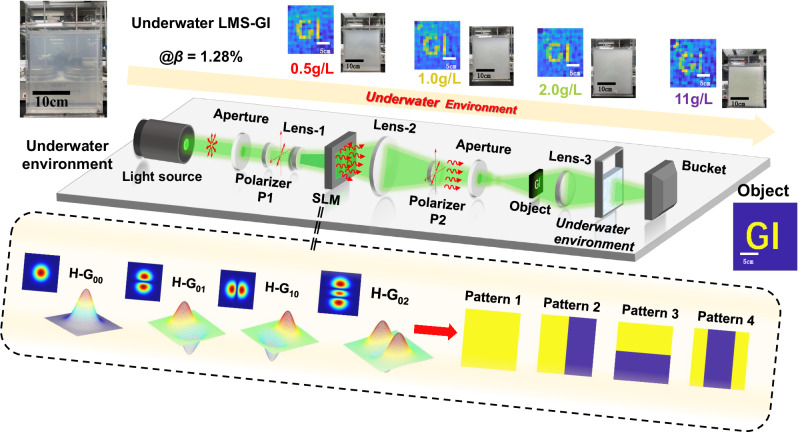
Scheme of the underwater experimental system. The proposed laser mode speckle pattern ghost imaging (LMS-GI) is a physical model based on the M^2^-sequence Hermitian-Gaussian (H-G) laser modes, and they are orthogonal.

The underwater experimental setup of LMS-GI is shown in Fig. 4. Using a laser with a collimation system (Fuzhe Laser Technology FU532D12-BD43) as the light source, a spatial light modulator (SLM) (UPOLabs RSLM1024) was used to generate computer-controlled laser mode patterns. The power meter (OPHIR PD300-3W-V1 and OPHIR StarLite) acted as a bucket detector and was recorded by the computer. The object under test was placed at the “object” shown in Fig. 4. The focal length of lens 1 was 10 cm, the focal length of lens 2 was 15 cm, and the focal length of lens 3 was 10 cm. The dimensions of the pool were 21 cm × 31.5 cm × 4.1 cm. We added 1.0 g, 2.0 g, 4.0 g, 6.0 g, 11 g, and 22 g of milk powder to 2 L of water, and then stirred the solution using a MeiYingPu H05-1 constant temperature magnetic stirrer to form uniformly turbid water. The concentrations were as follows: 0.5 g/L, 1.0 g/L, 2.0 g/L, 3.0 g/L, 5.5 g/L, and 11 g/L, respectively. The milk powder used had a protein content of 21.2 g/100 g and a fat content of 19.3 g/100 g. At concentrations greater than 1 g/L, conventional imaging methods using CCD/CMOS became ineffective.

**Fig. 5:**
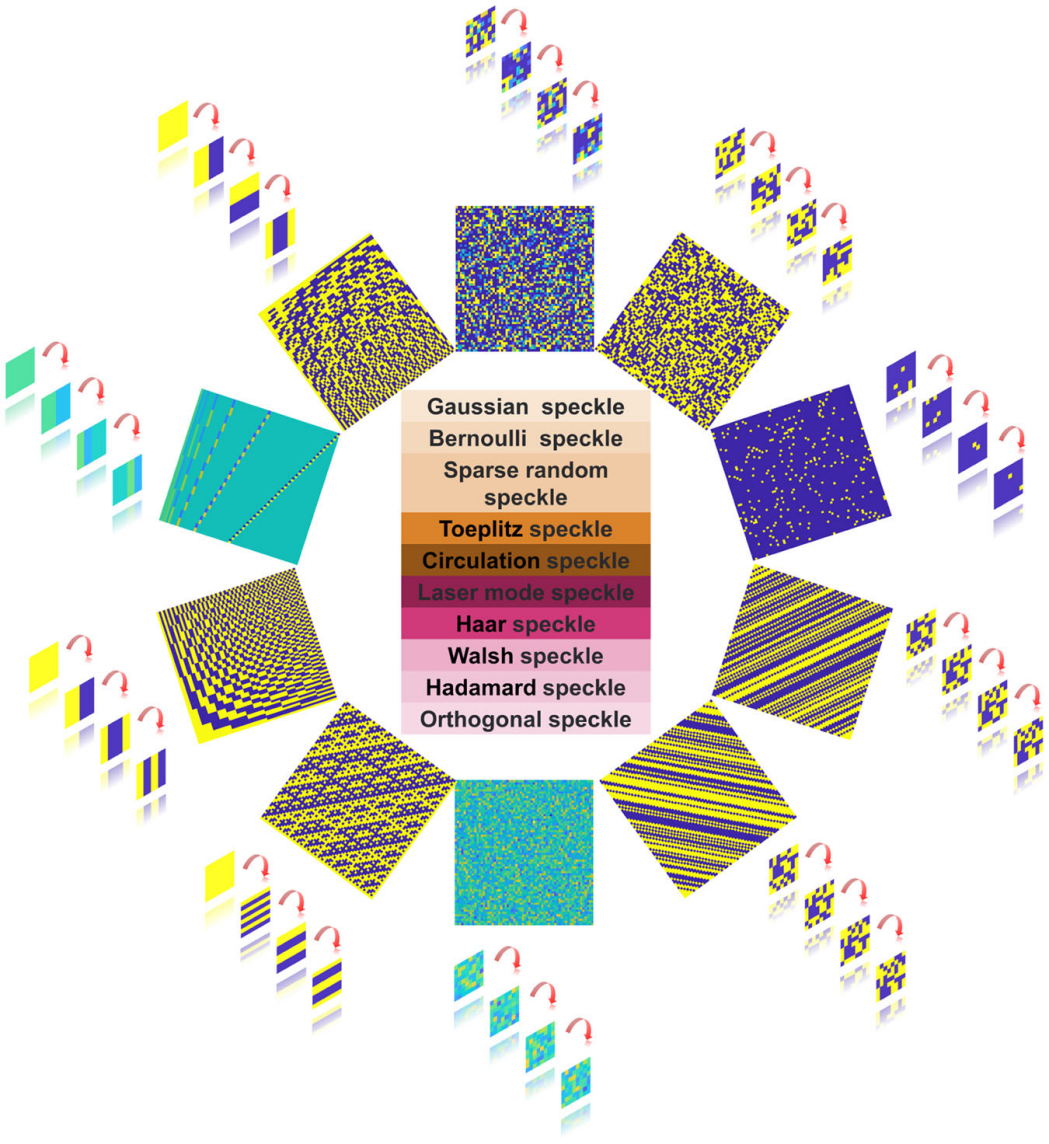
A partial list of speckle pattern matrices. The inner ring displayed the distribution of the speckle pattern matrix, while the outer ring showed the speckle patterns for each projection.

Figure 5 displays a partial list of the random speckle pattern matrices and orthogonal speckle pattern matrices. The inner part displayed the distribution of the speckle pattern matrix, while the outer part showed the speckle patterns for each projection.

## Discussion

**Fig. 6:**
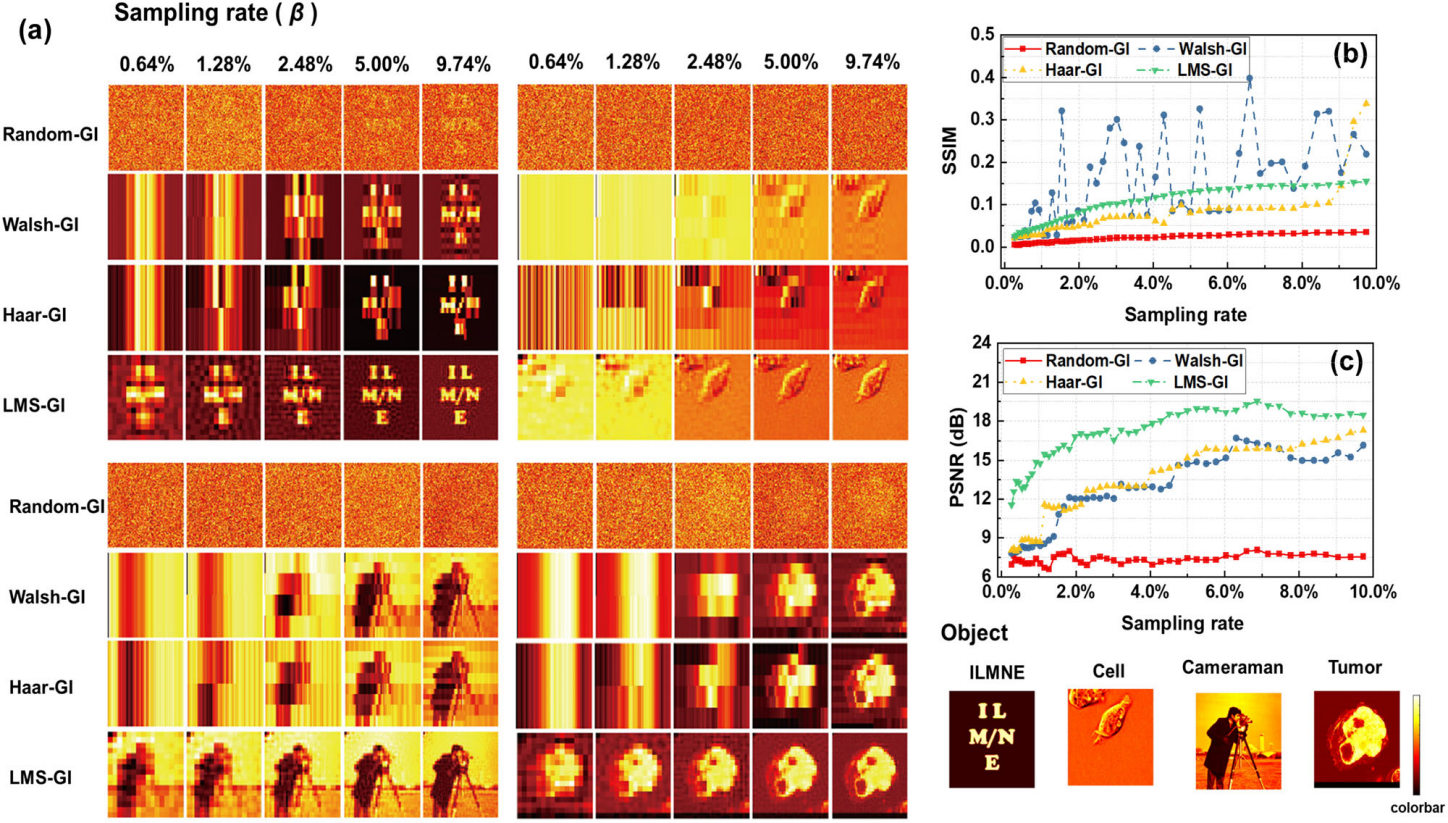
A comparison of simulated results for ghost imaging (GI). **a** Images reconstructed by Random speckle pattern GI (Random-GI), Walsh speckle pattern GI (Walsh-GI), Haar speckle pattern GI (Haar-GI), and Laser mode speckle GI (LMS-GI) at various sampling rates. There are four distinct types of objects being tested. “ILMNE” represents the digital type, “Cell” represents the cell type, “Tumor” represents the medical type, and “Cameraman” represents the human type. **b** The calculated structural similarity indicators (SSIM) of Random-GI (red square), Walsh-GI (blue circle), Haar-GI (yellow regular triangle), and LMS-GI (green inverted triangle) versus sampling rates for object “ILMNE”, respectively. **c** The peak signal-to-noise ratio (PSNR) values were calculated for Random-GI (red square), Walsh-GI (blue circle), Haar-GI (yellow regular triangle), and LMS-GI (green inverted triangle) versus sampling rates for object “ILMNE”, respectively.

We simulated four types of GIs for four types of objects with a distribution of $128\times 128$ pixels. Random speckle patterns, Walsh speckle patterns, and Haar speckle patterns were selected for comparison with our proposed LMS patterns. There are four different types of objects being tested. “ILMNE” represents the digital type, “Cell” represents the cell type, “Tumor” represents the medical type, and “Cameraman” represents the human type. The simulated results of random speckle pattern GI (Random-GI), Walsh speckle pattern GI (Walsh-GI), Haar speckle pattern GI (Haar-GI), and LMS-GI are shown in Fig. 6a. Due to the random nature of the random speckle patterns, the imaging quality for this type of GI is significantly lower than that for the other three GIs. Moreover, these three types of speckle patterns are orthogonal. Thus, perfect imaging can only be achieved at the sampling rate of $\beta =100$%. In addition, Walsh GI and Haar GI exhibit noticeable vertical stripe-like noise at low sampling rates. In contrast, LMS-GI can detect the overall trapped contour at a low sampling rate ($\beta =0.64$%). Furthermore, at a sampling rate of $\beta =2.48$%, the imaging results of LMS-GI fulfills the general requirements. At a slightly higher sampling rate of $\beta =5.00$%, details are clearly distinguishable in the “ILMNE”, “Cell”, and “Tumor”. In addition, the silhouette of the portrait in “Cameraman” is clearly visible, and the details of the camera stand are also distinct. The subjective judgment method alone is not sufficient to validate the excellent imaging quality of LMS-GI. Therefore, SSIM and PSNR analyses of the images were performed, as shown in Fig. 6b, c. In the SSIM shown in Fig. 6b, the LMS-GI value is significantly higher than that of Random-GI and Haar-GI. However, the Walsh-GI had a higher value than the LMS-GI in certain iterations of the experiment, which can be attributed to the SSIM calculation method, which can be attributed to the SSIM calculation method. In combination with the PSNR shown in Fig. 6c, the significant advantage of LMS-GI over the other GI methods at sampling rates below 10.0% is evident.

**Fig. 7:**
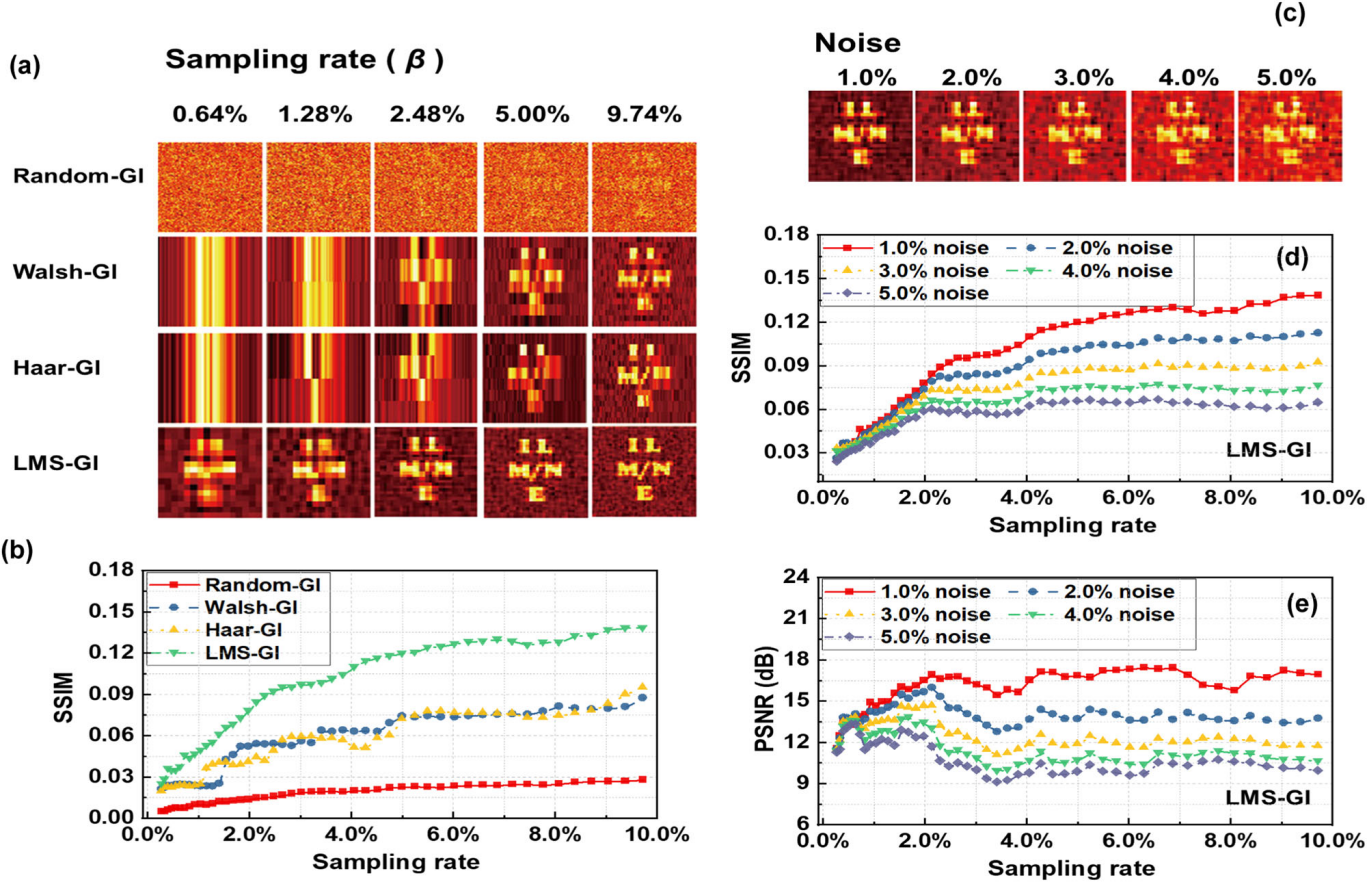
Simulated results of ghost imaging (GI) at various levels of relative random noise. **a** Images were reconstructed using Random speckle pattern GI (Random-GI), Walsh speckle pattern GI (Walsh-GI), Haar speckle pattern GI (Haar-GI), and Laser mode speckle pattern ghost imaging (LMS-GI) at various sampling rates, when a relative random noise of 1.0%. **b** The Structural Similarity (SSIM) indicators were calculated for Random-GI (red square), Walsh-GI (blue circle), Haar-GI (yellow regular triangle), and LMS-GI (green inverted triangle) versus sampling rates at 1.0% relative random noise. **c** The images were reconstructed by LMS-GI at a sampling rate of 2.48% with 1.0% to 5% relative random noise. Calculated the SSIM (**d**) and the peak signal-to-noise ratio (PSNR) (**e**) of LMS-GI versus sampling rates at 1.0% (red square), 2% (blue circle), 3% (yellow regular triangle), 4% (green inverted triangle), and 5% (gray diamond) relative random noise levels. The object is “ILMNE”.

To judge the imaging robustness, we added 1.0% relative random noise to the bucket of these four types of GI, and the imaging results are shown in Fig. 7a. We added 1.0% random wave noise by averaging the received values of the bucket detector, using a multiplier operation and a random function. Combined with the data shown in Fig. 7b, it can be seen that the image quality of LMS-GI was the best at the same sampling rate, indicating its relatively superior robustness compared to the other types of GI. At this time, 1.0%, 2.0%, 3.0%, 4.0%, and 5.0% relative random noise were added to the bucket detector of LMS-GI. The imaging results at a sampling rate of 2.48% are shown in Fig. 7c. It can be seen that even with noise, the imaging quality of LMS-GI was still better than the other three ideal imaging results without noise when the sampling rate was the same. By combining the SSIM and PSNR in Fig. 7d, e, it can be observed that LMS-GI was impacted by noise, leading to a decrease in its imaging quality as the level of noise increased. However, its robustness remained reliable.

**Fig. 8:**
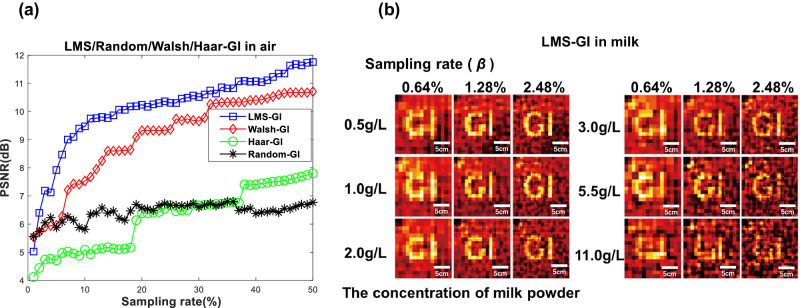
Experimental results of ghost imaging (GI). **a** Peak signal-to-noise ratio (PSNR) indicators versus sampling rates for laser mode speckle GI (LMS-GI, blue square), Walsh speckle pattern GI (Walsh-GI, red diamond), Haar speckle Pattern GI (Haar-GI, green circle), and Random speckle pattern GI (Random-GI, black star) in air. **b** Images reconstructed by LMS-GI at various sampling rates in turbid water.

We conducted Random-GI, Walsh-GI, Haar-GI, and LMS-GI experiments in both air and milk, as shown in Fig. 8. The presence of laser and bucket detection fluctuations during the experiment resulted in uncertain random values and positions, leading to experimental results that deviated from the ideal simulated results (Fig. 6c). Overall, the imaging quality of LMS-GI was much better than that of Random-GI, Walsh-GI, and Haar-GI. In general, when using LMS-GI, the imaging quality was good when the sampling rate was 10.0% or less. When the sampling rates were 0.64%, 1.28%, and 2.48%, the results of the turbid underwater LMS-GI experiment are shown in Fig. 8b. The concentrations of turbid water were: 0.5 g/L, 1.0 g/L, 2.0 g/L, 3.0 g/L, 5.5 g/L, and 11 g/L. When a CCD/CMOS was used for imaging, there was a blur when the turbid water concentration was 0.5 g/L, and it was almost invisible when the turbid water concentration was 1.0 g/L. The imaging quality of LMS-GI was high when the turbid water concentration was less than 1.0 g/L. When the concentration exceeded 1.0 g/L, the imaging quality decreased, but it was still possible to reconstruct the image. It can be seen that LMS-GI was still able to produce images at a low sampling rate of 0.64% in turbid water. Furthermore, the sampling rate was significantly lower than that of GI using compressed-sensing technology, and even lower than that of most deep-learning GI methods.

Table 1 provides a comparison of LMS-GI with underwater imaging GI techniques used by other researchers. The turbid water used by researchers is usually made by adding china clay or milk to water. When using china clay as the additive, the unit of turbidity measurement is Nephelometric Turbidity Unit (NTU) (1 NTU = 1 mg/L SiO_2_). When milk is added, the unit of turbidity used is Formazin Turbidity Unit (FTU), with Formazin used as the standard liquid. In this study, the instructions for the milk powders indicated that conventional milk is prepared by reconstituting 35 grams of milk powder in 200 mL of water. The ratio of milk powder to water used in the above study was converted to the milk-to-water ratio used by other researchers. The results ranged from 1:1000 to 1:7, covering both low-turbidity and high-turbidity areas. According to the experimental results, it can be seen that LMS-GI can be used not only in a low-turbidity underwater environment but also in a high-turbidity underwater environment. Compared to the sampling rates used by other researchers, our sampling rate of 2.48% is not the minimum value. However, the LMS-GI’s correlation algorithm is more convenient and has a lower computation time compared to GI methods using compressed-sensing technology.

**Table 1:** Comparison of laser mode speckle pattern ghost imaging (LMS-GI) with other ghost imaging techniques for turbid underwater imaging

Year	Algorithm	Water quality (turbidity)	Sampling rate
2017^38^	correlation calculation	china clay (80 NTU)	-
2019^39^	correlation calculation	black ink (0.4 mL)	-
2020^40^	correlation calculation	milk (milk: water = 1:1500, 32 FTU)	1
2021^41^	correlation calculation	kaolin clay powder (80 NTU)	20%
2021^10^	deep learning	milk (milk: water < 1:6.84)	20%
2021^11^	compressed sensing	milk (milk: water = 1:9)	2%–5%
Our LMS-GI method	correlation calculation	milk (milk: water < 1:7)	0.64%–2.48%

## Conclusions

In this study, we have proposed orthogonal speckle patterns based on a physical model of *M*^2^-ordered H–G laser mode patterns and applied them to GI. Simulated and experimental results have demonstrated that the LMS-GI can achieve ultra-low sampling imaging. When the sampling rate was 5%, we achieved high-quality imaging with the PSNR reaching 19 dB. However, at a sampling rate of 0.64%, a rough contour was able to be formed. The LMS-GI demonstrates strong robustness and can be used for imaging in turbid underwater environments. Compared to conventional GI and CCD/CMOS imaging, LMS-GI’s imaging quality is significantly improved. As a result, LMS-GI can be used as an imaging solution in challenging liquid environments, including turbid liquids, inclement weather, and biological tissue fluids.

## Methods

### Ghost imaging

The object has a resolution of ${n}_{1}\times {n}_{2}$, and the transmission distribution of the object is $T(x,y)$, where $x=1,2,{{{{\mathrm{..}}}}}.,{n}_{1}$,$y=1,2,{{{{\mathrm{..}}}}}.,{n}_{2}$ which can be converted to a matrix of $n\times 1$ dimension $X$, where $n={n}_{1}{n}_{2}$. The resolution of each scattergram is ${n}_{1}\times {n}_{2}$, the amount of projected speckle data is $m$, and the light intensity distribution of the *t-*th speckle pattern is $I(x,y,t)$, where $t=1,2,{{{{\mathrm{..}}}}}.,m$, converting the array of speckle patterns into $\varPhi$, which is a matrix of $m\times n$ dimension. And the corresponding *t*-h bucket detector value is:

13 $$B(t)=\iint I(x,y,t)T(x,y){{{{{\rm{dxdy}}}}}}$$

Form the light intensity collected by a bucket detector into a $m\times 1$ matrix $Y$. The imaging process of GI is:

14 $$Y=\varPhi X$$

The mean value of the *m* bucket detectors is:

15 $$\langle B\rangle =\frac{1}{m}\mathop{\sum }\limits_{t=1}^{m}B(t)$$

The mean value of the speckle patterns is:

16 $$\langle I\rangle =\frac{1}{m}\mathop{\sum }\limits_{t=1}^{m}I(x,y,t)$$

Using the second-order correlation for the calculation, the GI is obtained as:

17 $$G(x,y)=\frac{1}{m}\mathop{\sum }\limits_{t=1}^{m}\{[B(t)-\langle B\rangle ][I(x,y,t)-\langle I\rangle ]\}$$

### Statistics and reproducibility

In Fig. 7, the bucket detector updates its value every 1 s, with 0.5 s allocated for averaging to ensure data reliability.

### Laser mode evaluation criteria *M*^2^

The *M*^2^ factor has become one of the most widely accepted parameters for evaluating beam quality. The beam radii of the ${{{{{{\rm{H}}}}}}-{{{{{\rm{G}}}}}}}_{mn}$ modes in the *x* and *y* directions are:

18 $${w}_{x,mn}^{2}(z) 	 =\frac{4{\int }_{-\infty }^{+\infty }{x}^{2}{{{{{{\rm{H}}}}}}}_{m}^{2}\left(\frac{\sqrt{2}x}{{w}_{{{{{{\rm{os}}}}}}}}\right){{{{{{\rm{e}}}}}}}^{-\frac{2{x}^{2}}{{w}_{{{{{{\rm{os}}}}}}}^{2}}}{{{{{\rm{d}}}}}}x}{{\int }_{-\infty }^{+\infty }{{{{{{\rm{H}}}}}}}_{m}^{2}\left(\frac{\sqrt{2}x}{{w}_{{{{{{\rm{os}}}}}}}}\right){{{{{{\rm{e}}}}}}}^{-\frac{2{x}^{2}}{{w}_{{{{{{\rm{os}}}}}}}^{2}}}{{{{{\rm{d}}}}}}x}\\ 	 =(2m+1){w}_{{{{{{\rm{os}}}}}}}^{2}(z)\\ {w}_{y,mn}^{2}(z) 	 =\frac{4{\int }_{-\infty }^{+\infty }{x}^{2}{{{{{{\rm{H}}}}}}}_{n}^{2}\left(\frac{\sqrt{2}y}{{w}_{{{{{{\rm{os}}}}}}}}\right){{{{{{\rm{e}}}}}}}^{-\frac{2{y}^{2}}{{w}_{{{{{{\rm{os}}}}}}}^{2}}}{{{{{\rm{d}}}}}}y}{{\int }_{-\infty }^{+\infty }{{{{{{\rm{H}}}}}}}_{n}^{2}\left(\frac{\sqrt{2}y}{{w}_{{{{{{\rm{os}}}}}}}}\right){{{{{{\rm{e}}}}}}}^{-\frac{2{y}^{2}}{{w}_{{{{{{\rm{os}}}}}}}^{2}}}{{{{{\rm{d}}}}}}y}\\ 	 =(2n+1){w}_{{{{{{\rm{os}}}}}}}^{2}(z)$$

The beam radius of the ${{{{{{\rm{H}}}}}}-{{{{{\rm{G}}}}}}}_{mn}$ mode in *r* radial direction is:

19 $${w}_{r,mn}^{2}(z) 	 =\frac{4{\int }_{-\infty }^{+\infty }{\int }_{-\infty }^{+\infty }\left({x}^{2}+{y}^{2}\right){{{{{{\rm{H}}}}}}}_{m}^{2}\left(\frac{\sqrt{2}x}{{w}_{{{{{{\rm{os}}}}}}}}\right){{{{{{\rm{H}}}}}}}_{n}^{2}\left(\frac{\sqrt{2}y}{{w}_{{{{{{\rm{os}}}}}}}}\right){{{{{{\rm{e}}}}}}}^{-\frac{2({x}^{2}+{y}^{2})}{{w}_{{{{{{\rm{os}}}}}}}^{2}}}{{{{{\rm{d}}}}}}x{{{{{\rm{d}}}}}}y}{{\int }_{-\infty }^{+\infty }{\int }_{-\infty }^{+\infty }{{{{{{\rm{H}}}}}}}_{m}^{2}\left(\frac{\sqrt{2}x}{{w}_{{{{{{\rm{os}}}}}}}}\right){{{{{{\rm{H}}}}}}}_{n}^{2}\left(\frac{\sqrt{2}y}{{w}_{{{{{{\rm{os}}}}}}}}\right){{{{{{\rm{e}}}}}}}^{-\frac{2({x}^{2}+{y}^{2})}{{w}_{{{{{{\rm{os}}}}}}}^{2}}}{{{{{\rm{d}}}}}}x{{{{{\rm{d}}}}}}y}\\ 	 =2(m+n+1){w}_{{{{{{\rm{os}}}}}}}^{2}(z)$$

The waist half-width and far-field divergence half-angle of the fundamental mode Gaussian beam are set as ${w}_{0}$ and ${\theta }_{0}$, and

20 $${w}_{0}{\theta }_{0}=\frac{\lambda }{{{{{{\rm{\pi }}}}}}}$$

The half-widths of ${{{{{{\rm{H}}}}}}-{{{{{\rm{G}}}}}}}_{mn}$ bundle waist in the *x* direction, *y* direction, and *r* radial direction are:

21 $$\left\{\begin{array}{c}{w}_{0x,mn}^{2}=(2m+1){w}_{o}^{2}\hfill\\ {w}_{0y,mn}^{2}=(2n+1){w}_{o}^{2}\hfill\\ {w}_{0r,mn}^{2}=2(m+n+1){w}_{o}^{2}\end{array}\right.$$

The far-field diverging half-angles of ${{{{{{\rm{H}}}}}}-{{{{{\rm{G}}}}}}}_{mn}$ in the *x* direction, *y* direction, and *r* radial direction are:

22 $$\left\{\begin{array}{c}{\theta }_{x,mn}=\mathop{{{{{\mathrm{lim}}}}}}\limits_{z\to \infty }\frac{{w}_{x,mn}(z)}{z}=\sqrt{2m+1}\mathop{{{{{\mathrm{lim}}}}}}\limits_{z\to \infty }\frac{w(z)}{z}\hfill\\ =\sqrt{2m+1}\frac{\lambda }{{{{{{\rm{\pi }}}}}}{w}_{0}}=\sqrt{2m+1}{\theta }_{0}\hfill\\ {\theta }_{y,mn}=\mathop{{{{{\mathrm{lim}}}}}}\limits_{z\to \infty }\frac{{w}_{y,mn}(z)}{z}=\sqrt{2n+1}\mathop{{{{{\mathrm{lim}}}}}}\limits_{z\to \infty }\frac{w(z)}{z}\hfill\\ =\sqrt{2n+1}\frac{\lambda }{{{{{{\rm{\pi }}}}}}{w}_{0}}=\sqrt{2n+1}{\theta }_{0}\hfill\\ {\theta }_{r,mn}=\mathop{{{{{\mathrm{lim}}}}}}\limits_{z\to \infty }\frac{{w}_{r,mn}(z)}{z}=\sqrt{(2m+1)(2n+1)}\mathop{{{{{\mathrm{lim}}}}}}\limits_{z\to \infty }\frac{w(z)}{z}\hfill\\ =\sqrt{(2m+1)(2n+1)}\frac{\lambda }{{{{{{\rm{\pi }}}}}}{w}_{0}}=\sqrt{(2m+1)(2n+1)}{\theta }_{0}\end{array}\right.$$

The *M*^2^ factors in the *x* direction, *y* direction, and *r* radial direction of ${{{{{{\rm{H}}}}}}-{{{{{\rm{G}}}}}}}_{mn}$ can be expressed as:

23 $$\left\{\begin{array}{c}{M}_{x}^{2}=\frac{{{{{{\rm{\pi }}}}}}}{\lambda }{w}_{0x}{\theta }_{x}=\frac{{{{{{\rm{\pi }}}}}}}{\lambda }\sqrt{2m+1}{w}_{0}\sqrt{2m+1}{\theta }_{0}=2m+1\hfill\\ {M}_{y}^{2}=\frac{{{{{{\rm{\pi }}}}}}}{\lambda }{w}_{0y}{\theta }_{y}=\frac{{{{{{\rm{\pi }}}}}}}{\lambda }\sqrt{2n+1}{w}_{0}\sqrt{2n+1}{\theta }_{0}=2n+1\hfill\\ {M}_{r}^{2}=\frac{{{{{{\rm{\pi }}}}}}}{\lambda }{w}_{0r}{\theta }_{r}=\frac{{{{{{\rm{\pi }}}}}}}{\lambda }\sqrt{(2n+1)(2m+1)}{w}_{0}\sqrt{(2n+1)(2m+1)}{\theta }_{0}\\ =2(m+n+1)\hfill\end{array}\right.$$

## Data Availability

The data that support the findings of this study are available from the corresponding author upon request.

## Code availability

All codes used in this study are available from the corresponding authors upon request.
